# Implementation of an Enhanced Recovery after Surgery Pathway for Transgender and Gender-Diverse Individuals Undergoing Chest Reconstruction Surgery: An Observational Cohort Study

**DOI:** 10.3390/jcm12227083

**Published:** 2023-11-14

**Authors:** Nelson J. Aquino, Susan M. Goobie, Steven J. Staffa, Elizabeth Eastburn, Oren Ganor, Cathie T. Jones

**Affiliations:** 1 Department of Anesthesiology, Critical Care and Pain Medicine, Boston Children’s Hospital, Boston, MA 02115, USA; 2 Center for Gender Surgery, Boston Children’s Hospital, Boston, MA 02115, USA

**Keywords:** transgender, enhanced recovery after surgery, gender-affirming chest reconstruction surgery

## Abstract

Enhanced Recovery After Surgery (ERAS) protocols are associated with improved clinical outcomes in cisgender breast surgery patients. However, a paucity of research exists regarding transgender and gender-diverse individuals (TGD) in the ERAS framework. The primary objective of this observational cohort study is to describe the implementation of a gender-affirming ERAS protocol and its relationship to hospital length of stay (LOS) in TGD patients following chest reconstruction surgery. The secondary aim is to identify intraoperative predictors of LOS and define variables associated with adverse outcomes. We identified 362 patients in three epochs: a traditional group (*n* = 144), a partial ERAS implementation group (*n* = 92), and an ERAS group (*n* = 126). Exploratory multivariable median regression modeling was performed to identify independent predictors of LOS. We report that the traditional group’s median hospital LOS was 1.1 days compared to 0.3 days in the ERAS group. Intraoperative tranexamic acid administration was associated with significantly shorter LOS (*p* < 0.001), reduced postoperative drainage (*p* < 0.001), and fewer returns to the operating room within 24 h (*p* = 0.047). Our data suggest that implementing a multimodal ERAS gender-affirming pathway was associated with improved patient-centered surgical outcomes such as decreased return to the operating room for hematoma evacuation, higher rates of discharge home, and reduced postoperative drainage output.

## 1. Introduction

Transgender and gender-diverse (TGD) individuals are a growing demographic globally. Recent studies estimate that approximately 1.4% (~300,000) of youth in the United States (ages 13–17) identify as transgender [1]. The number of TGD individuals presenting for gender-affirming care is increasing. Gender-affirming surgery (GAS) is associated with improved mental health outcomes and is an effective life-changing treatment for gender incongruence [2]. Chest reconstruction surgery is one of the most commonly performed gender-affirming surgical procedures pursued [3]. One study reported an increase in adolescent gender dysphoria diagnoses from 2016 to 2019, along with a 389% increase in masculinizing chest reconstruction procedures performed in the ambulatory setting using the Nationwide Ambulatory Surgery Sample (NASS) database [4].

Enhanced Recovery After Surgery (ERAS) protocols are patient-focused care pathways developed by multidisciplinary health care teams from existing evidence-based literature and expert consensus, together with a consideration for institutional practice and culture. Previous studies suggest that ERAS pathways in cisgender breast surgery patients are associated with improved clinical outcomes and reduced length of stay (LOS) compared to conventional care [5,6,7,8]. Published evidence of gender-affirming chest reconstruction ERAS protocols is lacking, as are data on perioperative outcomes in the TGD population. There is emerging literature considering the biopsychosocial factors for TGD individuals in the perioperative environment [9]. With the paucity of clinical studies on gender-diverse young adult patients having gender-affirming surgery, evaluating perioperative patient-centered outcomes for this population may improve the delivery of safe, culturally sensitive, and evidenced-based care. Furthermore, these data may support future consensus on gender-affirming ERAS pathways and mitigate health care discrimination in the perioperative environment.

The primary aim of this study is to compare the hospital LOS between three groups of gender-diverse patients undergoing masculinizing chest reconstruction surgery by a single surgeon: a traditional group, a partial ERAS implementation group, and an ERAS group (details of these groups are described under “Methods”). The secondary aims are to identify intraoperative predictors of LOS and to compare the groups with regards to administration of intraoperative tranexamic acid (TXA), volume of 24 h postoperative Jackson Pratt (JP) drain output, and other specific adverse events (hematoma, seroma, cellulitis, and return to operating room).

## 2. Materials and Methods

This is a retrospective observational cohort study of TGD individuals who underwent elective gender-affirming chest reconstruction surgery from January 2017 to April 2022 in a single tertiary center. The local hospital institutional review board (IRB) approved the retrospective study (IRB-P00041762), which was exempt from informed consent. The data were gathered electronically by the anesthesia department research team who were not aware of the study aims. The patients included in the study were identified by the International Classification of Diseases (ICD)-10-code F.64 for gender dysphoria. The following Current Procedural Terminology (CPT) codes were included: bilateral mastectomy, nipple areola complex reconstruction, breast reduction, and nipple grafting. Gynecomastia repair, breast augmentation, and cisgender breast reduction patients were excluded from the data collection due to the difference in plastic surgeons and surgical techniques.

This led to a convenience sample of 362 gender-affirming chest reconstruction cases included in this study. The three epochs studied included a traditional group, partial ERAS implementation group and ERAS group. The data were extracted from a retrospective chart review of patient electronic medical records and anesthesia databases. The demographic data collected included age, weight, body mass index (BMI), the American Society of Anesthesiologists Physical Status (ASA-PS) classification, and gender identity. The perioperative characteristics identified were discharge plan, documented adverse events (hematoma, seroma, cellulitis, inpatient PONV incidence, return to operating room), and hospital LOS in days. The perioperative characteristics were identified in anesthesia, surgical, nursing, and EMR documentation. Post-anesthesia care unit (PACU) pain scores (numerical pain rating scale (NPRS)) were collected, and inpatient NPRS scores were identified.

### 2.1. Framework

In 2019, an anesthesiology subspecialty care service within the Department of Anesthesiology, Critical Care and Pain Medicine at Boston Children’s Hospital evolved to respond to an increase in transgender patients entering the perioperative environment for non-gender-affirming and gender-affirming procedures. The specialty anesthesiology team of anesthesiologists and certified registered nurse anesthetists (CRNAs), known as the Gender Affirming Surgical Perioperative Program (GASPP), established best practice clinical perioperative management recommendations for gender-affirming surgeries. Application of evidence-based principles and expert consensus guidelines, together with improvement in anesthesia and surgical techniques, led to the development of a patient-focused ERAS pathway. This process included a systemized review of published cisgender breast reconstruction ERAS pathways, together with experiential knowledge gained from chest reconstruction cases from 2019 to 2020. The GASPP team members critically evaluated outcomes which led to the formalization of a standardized multimodal gender-affirming chest reconstruction ERAS protocol.

### 2.2. Interventions

Initial clinical anesthesia perioperative guidelines for chest reconstruction surgery were posted on the departmental internal website, and its implementation was at the discretion of the anesthesiology team on the day of surgery. Key ERAS recommendations focused on mitigation strategies to reduce postoperative nausea, vomiting, pain, and coughing, as well as to reduce the risk of postoperative hematoma formation. In addition, team members demonstrated a preliminary case series by successively capturing all the chest reconstruction cases for one year to evaluate patient demographics and perioperative outcomes. Interdisciplinary collaboration between surgical, anesthesiology, and nursing teams supported open communication, advanced care coordination, and attention to evolving perioperative outcomes to create a patient-focused ERAS protocol. See Figure S1: Gender-Affirming Chest Reconstruction ERAS pathway.

### 2.3. Traditional Group

The traditional group started from the inception of the institutional gender surgery program on 1 January 2017 and ended on 31 May 2020. Chest reconstruction cases reviewed during this phase included randomly assigned anesthesiology clinicians (anesthesiologists, trainees, and CRNAs) with anesthesia management at the discretion of the clinicians. In November 2019, formalization of the anesthesiology specialty team initiated consistent participation in chest reconstruction cases. Utilizing both identification of International Classification Codes (ICD) for gender dysphoria and procedural chest reconstruction codes, the team administrator assigned GASPP team members to cases in the main hospital and the ambulatory setting.

### 2.4. Partial ERAS Implementation Group

The partial ERAS implementation group started on 1 June 2020 and ended on 28 February 2021. The partial ERAS implementation group included specialty anesthesiology team participation in chest reconstruction cases. Members facilitated the development of clinical anesthesia management guidelines (non-ERAS) based on existing published evidence of breast reconstruction in cisgender adults and experiential exposure. The team’s preliminary clinical anesthesia guidelines evolved into the departmental “un-official” or “partial” ERAS pathway during this period. Gender-affirming care considerations for TGD individuals (self-identification, gender-identify fields, and psychosocial factors) along with factors that impact anesthesia management and perioperative planning were included in chest reconstruction surgery guidelines. Monthly team meetings and consistent surgeon communication allowed for tailoring guidelines to mitigate adverse events and support improved outcomes during this phase. For example, remifentanil was implemented as an adjunct to mitigate coughing on emergence and prevent potential hematoma formation [10,11]. In addition, after increasing cases of return to the operating room for hematoma evacuation, TXA was implemented in the anesthesia management due to previous studies of TXA demonstrating reduced blood loss [12,13].

### 2.5. ERAS Group

**Figure 1:**
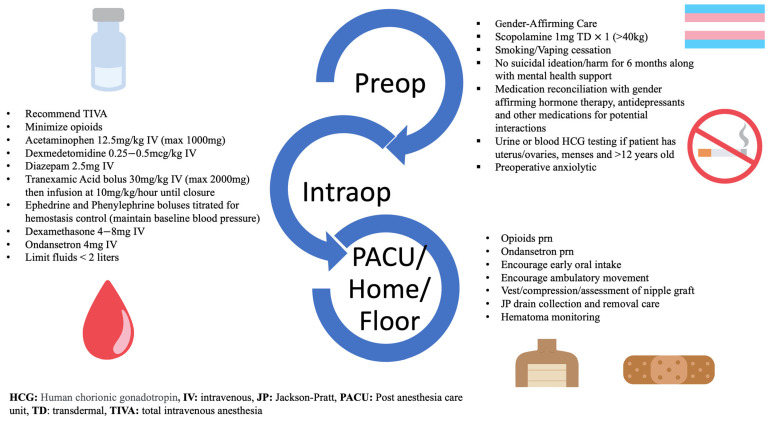
Highlights of the Enhanced Recovery After Surgery pathway.

The ERAS group phase started on 1 March 2021 and the case review of patients ended on 15 April 2022. After undergoing a Departmental review, the ERAS pathway was officially approved and formalized on 1 March 2021. All cases reported during this phase involved adherence to gender-affirming care, psychosocial care optimization, measures taken to prevent postoperative nausea and vomiting (PONV), and hemostasis management, followed by a multimodal pain regimen. The ERAS protocol outlined specific biopsychosocial considerations to optimize anesthesia management based on each TGD individual’s medical and surgical histories, including mental health concerns. Highlights of the ERAS protocol for gender-diverse individuals undergoing chest reconstruction surgery are described in Figure 1. GASPP team members educated anesthesiology staff, trainees, nurses, and multidisciplinary members on the protocol while soliciting feedback and expectations for applying it to clinical practice. In addition, to facilitate the implementation of the ERAS protocol, team members offered patients the option for communication and email consultation before the day of surgery.

### 2.6. Primary and Secondary Outcome Measures

Based on previous ERAS studies, the primary outcome selected for the study is the hospital length of LOS, defined as the time from admission to discharge from the hospital in days [5]. All records were audited and verified for accurate discharge status based on the electronic medical record documentation. Justification of hospital LOS was based on several challenges of confounding control, such as patient-specific factors, postoperative disposition criteria, perioperative adverse events reported, the COVID-19 pandemic, evolving anesthesia management, and research gaps in gender-affirming anesthesia clinical practices. Therefore, multivariable median regression modeling was performed to identify hypothetical independent predictors of LOS.

Yearly trends of tranexamic acid administration (from 2017–2022) and 24 h Jackson-Pratt (JP) inpatient drainage output were collected to compare perioperative bleeding outcomes in two cohorts: the TXA group receiving an intraoperative TXA bolus (30 mg/kg) and infusion (10 mg/kg/h), and the group not receiving TXA administration [12]. JP drainage output was collected prior to discharge home from the post-anesthesia care unit (PACU) and inpatient 24 h drainage output was collected from PACU and during inpatient overnight stay. For patients who were discharged home on the same day of surgery, the plastic surgery team recorded 24 h JP drainage output on a tracking form that was scanned to the electronic medical record. Missing data were denoted by different denominators presented within subgroups for categorical variables.

### 2.7. Statistical Analysis

Continuous data are presented as median (interquartile range) and categorical data are presented as frequency (percent). Data are summarized overall and by epoch group. Multivariable median regression modeling was performed to identify significant independent predictors of hospital LOS in an exploratory analysis of baseline and intraoperative factors, with results presented as adjusted coefficients with 95% confidence intervals and *p* values. Multivariable logistic regression analysis was implemented to explore predictors of the composite outcome of hematoma or return to OR, with results presented as adjusted odds ratios with 95% confidence intervals and *p* values. Bivariate group comparisons were performed using the Chi-square test, Fisher’s exact test, or the Wilcoxon rank sum test, as appropriate. A two-tailed *p* < 0.05 was considered statistically significant in the exploratory analysis of predictors of LOS. All statistical analyses were performed using Stata (version 16.1, StataCorp LLC, College Station, TX, USA).

## 3. Results

### 3.1. Demographics and Patient Characteristics

A total of 362 patients underwent masculinizing chest reconstruction surgery (traditional group = 144; partial implementation ERAS group = 92; ERAS group = 126) over five years. The median age of patients was 18 years (IQR: 17, 21) with the youngest patient presenting at 15 years old with parental consent. Patients who were ASA-PS I and II constituted 95.3% of cases; 28.7% of the elective cases were performed at the primary hospital with 73.1% of the surgeries completed in the institutional ambulatory environment with overnight inpatient observation if necessary. A summary of demographics and patient characteristics is displayed in Table 1.

### 3.2. Group Implementation

The median hospital LOS was 1.1 days (interquartile range (IQR): 1, 1.2) in the traditional group, 0.3 days (IQR: 0.3, 0.6) in the partial ERAS implementation group, and 0.3 days (IQR: 0.2, 1) in the ERAS group. Adverse events were defined as hematoma, seroma, cellulitis, and return to the operating room. Adverse events were identified by the ICD 10 and CPT codes and by auditing each chart after discharge for complications and readmission. Some patients were documented as having more than one adverse event. A total of 35 adverse events were observed (traditional *n* = 17, partial ERAS *n* = 11, ERAS *n* = 7) consisting of 17 wound hematomas, 12 seromas, 10 cellulitis, and 7 cases returning to the operating room for hematoma evacuation. Ten cellulitis patients were identified who required fluid removal and antibiotic treatments after visits to the postoperative clinic or emergency room. Ten patients presenting with seromas required needle aspiration of fluid in the emergency department or during the postoperative clinic visit. In addition, 19 cases of inpatient PONV were reported and the incidence of PACU PONV was not collected due to a lack of documentation. See Table 2 for the primary outcomes analysis and adverse events. Since the aim of the study was to perform a descriptive analysis of the measurable variables in each epoch, we cannot include *p* values in Table 2.

### 3.3. Intraoperative Predictors of Length of Stay

Exploratory multivariable analysis of risk factors associated with hospital LOS is presented in Table 3. ASA-PS III was associated with longer LOS (adjusted coefficient = 0.35; 95% CI: 0.05, 0.65; *p* = 0.023), as well as peripheral nerve block (paravertebral blocks and/or catheters) (adjusted coefficient = 1.32; 95% CI: 0.79, 1.84; *p* < 0.001), and location at the main hospital site (adjusted coefficient = 0.17; 95% CI: 0.04, 0.3; *p* = 0.01). Administration of intraoperative TXA usage was associated with shorter LOS (adjusted coefficient = −0.68; 95% CI: −0.84, −0.53; *p* < 0.001).

### 3.4. Tranexamic Acid Comparisons

Table 4 summarizes intraoperative TXA administration, baseline factors, yearly trends of TXA usage, PACU to discharge JP drainage, inpatient 24 h JP drainage, and adverse outcomes. Patients receiving intraoperative TXA infusion were older (median = 19 years (IQR: 17, 22) vs. median = 18 years (IQR: 17, 20), *p* = 0.014). TXA administration was associated with higher rates of discharge home (162/239, 67.8%) for discharge disposition as compared to no intraoperative TXA administration (10/123, 8.1%) (*p* < 0.001). TXA administration was associated with decreased 24 h inpatient JP drainage (median = 0.82 mL/kg with TXA (IQR: 0.61, 1.17) versus 1.5 mL/kg without TXA (IQR: 1.06, 2.25); *p* < 0.001. In addition, TXA administration was associated with a lower volume of cases requiring return to the operating room within 24 h of surgery for hematoma evacuation (0.8%) compared to the absence of TXA use (5.1%) (difference = −4.3%, 95% CI: −8.3%, −0.1%); *p* = 0.047). There were no reported thromboembolic events throughout the study. Before the implementation of TXA in 2019, five out of seven patients returned to the OR for hematoma evacuation after non-surgical management (needle aspiration of increased fluid and dressing compression on chest) within 24 h. In 2020, two cases returned to the OR for hematoma/seroma evacuation after receiving intraoperative TXA. The surgeon elected to use conservative hematoma management in one patient, resulting in a return to the OR. In the second case, the patient returned to the OR for further JP drainage after emesis and implementation of compression dressing. From 2021, with the ERAS implementation of TXA, there were no reported cases of returning to the operating room within 24 h for hematoma evacuation.

### 3.5. Intraoperative Predictors of Hematoma or Return to OR

Exploratory multivariable analysis of risk factors associated with the composite outcome of hematoma or return to OR revealed that ASA-PS III was associated with increased odds of the composite outcome (adjusted odds ratio = 10.8; 95% CI: 1.09, 106.1; *p* = 0.042). Administration of intraoperative TXA was not significantly associated with hematoma or return to OR after adjusting for all other intraoperative factors (adjusted odds ratio = 1.34; 95% CI: 0.25, 7.18; *p* = 0.731).

## 4. Discussion

In this retrospective observational cohort study, we reviewed a comprehensive database to gain valuable insights into patient demographics, as well as intraoperative and postoperative variables that may influence the improvement of patient-centered surgical outcomes achieved through an ERAS pathway for gender-affirming chest reconstruction surgery. In an exploratory multivariable analysis of risk factors associated with hospital LOS days, the ERAS intervention of TXA infusion was the most important predictor of decreased LOS when compared to other intraoperative medications and interventions. In addition, intraoperative TXA infusion (as part of the ERAS protocol) was associated with a reduced return to operating room for hematoma evacuation in the 24 h period after surgery, higher rates of discharge home, and reduced PACU and inpatient JP drainage output.

Our findings are consistent with other published reports of improvements in patient outcomes utilizing ERAS pathways implemented for cisgender adults having breast reconstruction surgeries. In a comparative study of 330 microsurgical breast reconstruction patients describing three groups—traditional routine care (control), transitional cohort (partial implementation of ERAS), and ERAS group—hospital LOS was reduced by two days in the ERAS group compared to the traditional group (*p* < 0.0001) [5]. Two ERAS meta-analyses in the adult literature include observational and interventional studies suggesting decreased LOS, reduced readmission rates, risk stratification, cost effectiveness, improved patient experience, and improved perioperative outcomes in multiple disciplines, including anesthesiology and surgery [14,15]. Previous retrospective studies suggest the implementation of ERAS pathways in young adults undergoing idiopathic scoliosis surgery and laparoscopic gynecologic surgeries have impacted LOS, mobilization, and postoperative opioid consumption [16,17]. There continues to be a gap in age-specific (pediatric, adolescent, geriatric), emergent, and vulnerable cohort (gender-diverse, socioeconomic status) ERAS pathways in anesthesiology [14].

Furthermore, we report a reduced rate of return to the OR, while demonstrating no significant difference in rates of hematoma, seroma, or cellulitis when compared to routine care. In addition, administering intraoperative TXA was associated with shortened LOS, reduced JP drainage output, and a lower number of patients requiring the return to the OR for hematoma evacuation in 24 h (7 out of 362 cases). Utilizing the American College of Surgeons National Surgical Quality Improvement Program^®^ (ACS NSQIP) database from 2010–2017, Cuccolo et al. reported a rate of unplanned operation of 3.2% (19 out of 591 cases) secondary to hematoma (1.5%), abscess (0.7%), and bleeding (0.25%) in 591 transgender and non-binary individuals undergoing masculinizing chest reconstruction surgery [18]. In a multivariable regression analysis of intraoperative predictors of hematoma or return to OR, we report that ASA-PS III status is associated with an increased risk. In a retrospective data analysis of transgender individuals having gender-affirming chest reconstruction surgery, Cuccolo et al. report no independent risk factors for adverse outcomes and reasons for reoperation [18]. In a retrospective chart review of 679 female to male patients undergoing gender-affirming chest reconstruction mastectomies, McEvenue et al. report a total hematoma rate of 4.9%, seroma rate of 6.5%, and 1.6% reoperation rate for hematoma evacuation [19].

Our patient-centered ERAS protocol was developed to be consistent with similar breast reconstruction and microvascular ERAS pathways. It includes the ERAS Society consensus recommendations for breast reconstruction surgery, focusing on total intravenous anesthesia (TIVA), multimodal PONV prophylaxis, and pain management throughout the perioperative period [5,6,7,8]. Although the incidence is low, we report that the incidence of inpatient PONV was 12% lower in the partial ERAS and ERAS group, compared to the traditional group, which is consistent with similar studies in breast reconstruction ERAS pathways [5,6]. PONV reduction is likely due to recommended PONV prophylaxis interventions such as a scopolamine patch, dexamethasone, ondansetron, and a combination of TIVA techniques.

Our ERAS protocol differs from other ERAS pathways for breast surgery by including intravenous TXA administration for perioperative bleeding management. Furthermore, our findings agree with other studies on the potential benefit of TXA in reducing blood loss for cisgender breast reconstruction and microvascular surgeries [20,21]. This multimodal approach was associated with statistically and clinically significant reductions in JP drainage output (prior to discharge and inpatient 24 h) and fewer returns to the operating room in 24 h for hematoma evacuation. In our multivariable regression analysis, intravenous TXA infusion was independently associated with a reduction in hospital LOS for transgender individuals having gender-affirming chest reconstruction surgery.

We report that PACU pain NPRS scores were low (0–3) to medium (4–6), and inpatient numeric pain scale scores consistently remained below 3 out of 10 across all three epochs. The reported pain scores are consistent with results from a retrospective study comparing pain outcomes following mastectomy or bilateral breast reduction for transgender and cisgender patients who received pectoralis nerve blocks and in a comparative study of microsurgical breast reconstruction in adults [22]. Since our pain scores do not appear different to those achieved with routine use of regional anesthesia, our practice is to reserve regional anesthesia for those patients with specific comorbidities such as chronic pain. As a result of this practice, in our data, the use of regional anesthesia was associated with a longer LOS. In our ERAS pathway, regional anesthesia is recommended for patients with substance use disorders who are on chronic opioid agonists requiring opioid-sparing anesthesia management.

The implementation of evidence-based principles and expert consensus guidelines, combined with advancements in anesthesia and surgical techniques, has facilitated the establishment of a gender-affirming Enhanced Recovery After Surgery (ERAS) pathway at our institution. When comparing this pathway to previous ERAS oncologic breast reconstruction, it is essential to consider the differences between young and older adult populations. Differences include the presence of more coexisting morbidities such as morbid obesity, smoking history, and prior chemotherapy and radiation treatments in older adults, as well as psychosocial differences in the gender-diverse population [5,23].

### Limitations

Identifying which elements of our ERAS pathway individually contributed to improved patient outcomes and shortened hospital LOS is not possible due to the retrospective nature of the report and multimodal ERAS elements. Due to the temporal nature of the ERAS protocol implementation, our study did not aim to evaluate a causal relationship between our ERAS protocol and the measured outcome variables. We are unable to evaluate a causal relationship given the retrospective nature of the data and the difficulty with controlling for temporal effects with the given sample sizes. We aimed to describe the measured variables during each epoch and summarize the descriptive findings.

Our study has several limitations, particularly surrounding factors determining LOS. Due to the COVID-19 pandemic and the availability of inpatient hospital beds, there was a significant pivot in patient disposition from mostly admission (89.6% traditional) to mostly day surgery (69.1% ERAS). While there is a decrease in the LOS over our study period, we cannot directly attribute these changes to ERAS implementation because of the pandemic and scheduling limitations. In addition, the sample size of the partial implementation group was impacted by elective surgery scheduling restrictions caused by the COVID-19 pandemic. As we found no increased morbidity with earlier discharge, this continued through the full ERAS implementation.

As ERAS implementation proceeded, the Hawthorne effect is a noteworthy unmeasurable variable that may have influenced outcomes, particularly concerning the evolution over time in anesthesia and surgical management. Chest reconstruction procedures were performed in two different surgical centers: our main hospital and the ambulatory surgery center guided by surgical booking preference considering patient co-morbidities. Additional limitations of our study include it being a single-center study with one surgeon and with surgical practice evolution due to increased experience and time. Surgical techniques for chest reconstruction surgery were individualized and varied depending on the patient’s body characteristics, desires, and suitability; surgical management evolved throughout this study.

Additionally, as with any retrospective study, there may be variability in what is documented by different practitioners. However, all records were manually audited to confirm and validate the correctness of the perioperative, PACU, and inpatient data, including gender identity, diagnosis, type of surgery, medications, and adverse events (as reported using ICU-10 codes) within the Anesthesia Information Management System (AIMS) record and medical records. PACU documentation of PONV incidence was not consistently recorded and therefore was not included in the final data analysis.

In benign breast surgery, there is limited evidence on the independent association of intravenous TXA administration reducing hematoma formation and decreasing drain volume 24 h post surgery [20]. Due to the same-day discharge, a consistent measurement of drain volume within the PACU was available in the partial and complete ERAS implementation. Also, a notable limitation of this retrospective study is the possibility of selection bias and decreased elective procedures during the COVID-19 pandemic. Before 2019, TXA administration was not standard; it was at the discretion of the anesthesia team. In 2019, a TXA protocol was started due to the desire to minimize perioperative hemostatic bleeding. In addition, it is possible that clinical anesthesia and surgical practice (local anesthesia filtration and improved surgical end times) may have changed over time, and specific contributing variables may not have been accounted for. For example, to assess hemostasis before closing and to reduce the risk of hematoma formation, titrated doses of phenylephrine and/or ephedrine were administered to elevate blood pressure to the patient’s preoperative baseline values. Future robust studies may be warranted to evaluate TXA effectiveness on hemostatic-related outcomes for other gender-affirming surgical procedures.

## 5. Conclusions

Applying multimodal ERAS interventions to gender-affirming chest reconstruction surgery was associated with improved patient-centered surgical outcomes. Our study highlights that implementing an ERAS protocol as part of gender-affirming chest reconstruction surgery is both feasible and adaptable to gender-diverse young adults. In addition, intraoperative TXA administration in transgender and gender-diverse patients is associated with clinical advantages such as reduced LOS, improved discharge disposition to home, and reduced postoperative drainage output. Implementing a multimodal, patient-centered ERAS protocol as part of gender-affirming chest reconstruction surgery may be important in advancing anesthesia management and perioperative outcomes for the TGD population. Future prospective studies are warranted to confirm and evaluate which individual interventions contribute to delivering effective, safe, and affirming care for other gender-affirming procedures.

## Figures and Tables

**Table 1:** Demographics and patient characteristics.

Variable	Overall (*n* = 362)	Traditional Group (*n* = 144)	Partial ERAS Group (*n* = 92)	ERAS Group (*n* = 126)
Age (years)	18 (17, 21)	18 (17, 20)	19 (18, 22)	20 (17, 23)
Weight (kg)	71 (60, 86)	71 (60, 84)	69 (60, 87)	74 (62, 87)
BMI (kg/m^2^)	27 (23, 32)	26 (23, 31)	25 (22, 32)	27 (23, 32)
Gender Identity				
Non-binary	34 (9.4%)	8 (5.6%)	9 (9.8%)	17 (13.5%)
Transmasculine	328 (90.6%)	136 (94.4%)	83 (90.2%)	109 (86.5%)
Hospital Location				
Main Hospital	104 (28.7%)	30 (20.8%)	35 (38%)	39 (31%)
Ambulatory Center	258 (71.3%)	114 (79.2%)	57 (62%)	87 (69%)
ASA-PS				
I	77 (21.3%)	35 (24.3%)	18 (19.6%)	24 (19.1%)
II	268 (74%)	102 (70.8%)	70 (76.1%)	96 (76.2%)
III	17 (4.7%)	7 (4.9%)	4 (4.4%)	6 (4.8%)
Peripheral Nerve Block (Paravertebral block/catheters)	4 (1.1%)	3 (2.1%)	1 (1.1%)	0 (0%)
Discharge Disposition				
Discharged Home	172 (47.5%)	15 (10.4%)	70 (76.1%)	87 (69.1%)
Inpatient Floor	190 (52.5%)	129 (89.6%)	22 (23.9%)	39 (31%)

Continuous data are presented as median (interquartile range) and categorical data are presented as *n* (%). Abbreviations: ERAS, Enhanced Recovery after Surgery; BMI, Body Mass Index; ASA-PS, American Society of Anesthesiologists Physical Status. Non-binary—an umbrella term for people whose gender identity is neither male nor female. They may fall somewhere on the spectrum between male and female or have another gender entirely. Transmasculine—a person assigned female at birth with a more masculine gender identity, includes transgender men as well as non-binary individuals.

**Table 2:** Description of Primary and Secondary Outcomes.

Outcomes	Traditional Group (*n* = 144)	Partial ERAS Group (*n* = 92)	ERAS Group (*n* = 126)
Primary Outcome			
Hospital Length of Stay (days)	1.1 (1, 1.2)	0.3 (0.3, 0.6)	0.3 (0.2, 1)
Secondary Outcomes			
PACU Pain *^a^*	*n* = 141		
Low (0–3 NPRS score)	93 (66%)	65 (70.7%)	69 (54.8%)
Medium (4–6 NPRS score)	43 (30.5%)	22 (23.9%)	49 (38.9%)
High (7–10 NPRS score)	5 (3.6%)	5 (5.4%)	8 (6.4%)
Inpatient 24 h Pain Score (Numeric Pain Scale)	3 (2, 4)	2 (1, 4)	3 (2, 5)
Inpatient PONV	19 (13.2%)	1 (1.1%)	1 (0.8%)
Adverse Event	17 (11.8%)	11 (12%)	7 (5.6%)
Hematoma	8 (5.6%)	5 (5.4%)	4 (3.2%)
Seroma	4 (2.8%)	6 (6.5%)	2 (1.6%)
Cellulitis	8 (5.6%)	1 (1.1%)	1 (0.8%)
Return to OR	5 (3.5%)	2 (2.2%)	0 (0%)

Continuous data are presented as median (interquartile range) and categorical data are presented as *n* (%). *^a^* PACU Pain Scores: 3 cases “unable to answer”. Abbreviations: ERAS, Enhanced Recovery after Surgery; NPRS, Numeric Pain Rating Scale (range 0–10); PACU, Post-Anesthesia Care Unit; PONV, Postoperative Nausea and Vomiting.

**Table 3:** Multivariable median regression analysis of hospital length of stay (days).

Covariate	Adjusted Coefficient	95% CI	*p* Value
Age (years)	−0.01	(−0.02, 0.01)	0.519
Weight (kg)	0.002	(−0.002, 0.005)	0.341
Hospital Location Main Hospital (reference = Ambulatory Center)	0.17	(0.04, 0.3)	0.01 *
ASA-PS			
I	Reference		
II	0.01	(−0.12, 0.15)	0.83
III	0.35	(0.05, 0.65)	0.023 *
Peripheral Nerve Block (Paravertebral block/catheters)	1.32	(0.79, 1.84)	<0.001 *
Intraoperative Medications			
Acetaminophen Given	−0.1	(−0.55, 0.35)	0.657
Dexmedetomidine Given	0.05	(−0.08, 0.18)	0.475
Diazepam Given	0.01	(−0.11, 0.14)	0.825
Fentanyl Given	0.04	(−0.08, 0.16)	0.514
Hydromorphone Given	0.19	(−0.24, 0.63)	0.377
Ketamine Given	0.54	(0.29, 0.79)	<0.001 *
Morphine Given	0.32	(−0.28, 0.91)	0.295
Remifentanil Given	0.03	(−0.96, 1.03)	0.947
Nausea Drugs			
Dexamethasone Given	0.09	(−0.18, 0.36)	0.532
Haloperidol Given	0.46	(0.01, 0.91)	0.048 *
Ondansetron Given	−0.03	(−0.35, 0.29)	0.845
Scopolamine Given	−0.01	(−0.12, 0.11)	0.942
Intraoperative Infusions and Halogenated Agents			
Remifentanil Infusion Given	−0.12	(−1.12, 0.88)	0.813
Propofol Infusion Given	Cannot calculate-all patients received		
Sevoflurane given	−0.04	(−0.2, 0.12)	0.633
Isoflurane given	−0.25	(−0.71, 0.21)	0.292
Desflurane given	0.04	(−0.08, 0.16)	0.487
Tranexamic Acid Bolus given	−0.01	(−0.37, 0.34)	0.945
Tranexamic Acid Infusion given	−0.68	(−0.84, −0.53)	<0.001 *

* Statistically significant. Abbreviations: ASA-PS, American Society of Anesthesiologists Physical Status; CI, confidence interval.

**Table 4:** Intraoperative and Postoperative Data by Intraoperative TXA Administration.

Variable	Intraoperative TXA Infusion Given (*n* = 239)	No Intraoperative TXA Infusion Given (*n* = 123)	Difference (95% CI)	*p* Value
Age (years)	19 (17, 22)	18 (17, 20)	1 (0.2, 1.8)	0.014 *
Weight (kg)	71 (60.1, 86.8)	70.6 (59.8, 82.6)	0.4 (−5.1, 5.9)	0.886
ASA-PS				
I	43 (18%)	34 (27.6%)	N/A	0.065
II	186 (77.8%)	82 (66.7%)		
III	10 (4.2%)	7 (5.7%)		
Discharge Disposition				
Discharged Home	162 (67.8%)	10 (8.1%)	N/A	<0.001 *
Inpatient Floor	77 (32.2%)	113 (91.9%)		
Yearly Trends of TXA Administration				
2017	0 (0%)	4 (3.3%)	N/A	<0.001 *
2018	0 (0%)	37 (30.1%)		
2019	1 (0.4%)	77 (62.6%)		
2020	97 (40.6%)	3 (2.4%)		
2021	97 (40.6%)	2 (2.4%)		
2022	44 (18.4%)	0 (0%)		
PACU and Inpatient Drain Output within 24 h				
PACU JP Drainage Output prior to discharge home (mL/kg) *^a^*	0.23 (0, 0.53) *n* = 154	0.68 (0.18, 1.25) *n* = 5	−0.45 (−0.85, −0.05)	0.048 *
Inpatient 24-h JP Drainage Output (inpatient overnight) (mL/kg)	0.82 (0.61, 1.17) *n* = 85	1.5 (1.06, 2.25) *n* = 118	−0.68 (−0.92, −0.48)	<0.001 *
Adverse Events				
Hematoma	11 (4.6%)	6 (4.9%)	−0.3% (−4.9%, 4.3%)	0.999
Seroma	8 (3.4%)	4 (3.3%)	0.1% (−3.8%, 4%)	0.999
Cellulitis	4 (1.7%)	6 (4.9%)	−3.2% (−7.4%, 0.1%)	0.095
Return to operating room in 24 h	2 (0.8%)	5 (5.1%)	−4.3% (−8.3%, −0.1%)	0.047 *

Continuous data are presented as median (interquartile range) and categorical data are presented as *n* (%). *p* values were calculated using the Wilcoxon rank sum test, the Chi-square test, or Fisher’s exact test. * Statistically significant. *^a^* For patients discharged home (*n* = 159), there were *n* = 33 with scanned documents and *n* = 126 with no scanned documents. Abbreviations: CI, confidence interval; TXA, Tranexamic Acid; PACU, Post Anesthesia Care Unit; JP, Jackson-Pratt. N/A = not applicable.

## Data Availability

Data are contained within the article.

## Supplementary Materials

The following supporting information can be downloaded at: https://www.mdpi.com/article/10.3390/jcm12227083/s1, Figure S1: Gender-Affirming Chest Reconstruction ERAS pathway; Supplementary File S1: ERAS Document.
