# Supplementary material for: Implementation of an Enhanced Recovery after Surgery Pathway for Transgender and Gender-Diverse Individuals Undergoing Chest Reconstruction Surgery: An Observational Cohort Study
Source: J Clin Med. 2023 Nov 14;12(22):7083. doi: 10.3390/jcm12227083 (PMC10672389; doi:10.3390/jcm12227083)
Supplement: Supplementary file 1 [file jcm-12-07083-s001.zip › jcm-2647114-supplementary.pdf]

Enhanced Recovery after Surgery (ERAS) for Gender Affirming Chest Reconstruction Surgery

Preoperative Counseling and Education

1. Patients will be counseled regarding ERAS in Preop Clinic (if staying overnight) and Surgical Clinic. All patients should be familiar with our written and online materials to educate patients and providers about the ERAS protocol.

2. When booking these patients, the surgeon will indicate that this is an ERAS patient.

3. Patients will be counseled on timing of surgery, Body Mass Index (BMI), smoking/vaping cessation, and safe chest binding practices.[1–4]

4. Patients will be counseled on the importance of a supportive environment for postoperative recovery and expectations for pain management and surgical outcome.

5. Transgender patients have an increased rate of depression, anxiety, post-traumatic stress disorder, vaping, smoking and substance disorder.[5–7] A thorough history of substance use and medication history with particular attention to use of antidepressants (such as SSRIs) and possible interactions with anesthetic agents is important.[5,7–9]

6. All patients will meet with a dedicated social worker to ensure they have a therapist and have not engaged in self-harm, had psychiatric hospitalizations, or suffered from eating disorders in the last 6 months.[1]

7. The surgical team will use clinical judgment as well as World Professional Association of Transgender Health – Standard of Care (WPATH SOC) 8 guidelines (see references) to assist in finding appropriate timing of surgery alongside a multidisciplinary clinical team (primary gender team, surgeon, physician, physician assistant, social worker, and nursing).[10,11,12,13]

8. Clinicians should consider a phone call from the anesthesia team to the patient the day before surgery to address specific concerns related to anesthesia and provide some anxiety relief, as patients may have Generalized Anxiety Disorder and increased risk of behavior health concerns (suicidal harm/ideation, substance disorder) due to minority stress and health care discrimination.[5,7,8]

Preoperative (Day of Surgery)

1. Perioperative fasting guidelines as per institution's guidelines.

2. Obtain baseline laboratory tests based on physical assessment, abnormal findings and comorbidities.[5,7] Gender affirming hormone therapy (GAHT) is not used for contraception and may alter baseline laboratory values (hematocrit, hemoglobin and creatinine).[8]

3. Urine or blood human chorionic gonadotropin (HCG) is required unless patient has had a hysterectomy (see algorithm below). Patients are counseled about this by the surgical team. [5,7,8]

4. Careful reconciliation of medications due to potential interaction with anesthetics as many of these patients are on multiple medications with limited drug to drug interactions, including gender-affirming hormones (estradiol, progesterone, testosterone), antipsychotics, and beta blockers.[5,7,8] Consult gender team endocrinologist and/or surgeon for recommendations on GAHT.

5. Premedication with anxiolytic and/or adjuncts may require increased dosing due to significant anxiety and previous traumas.[2,5,7,8]

6. Collaboration with mental health provider, social worker, or child life specialist at bedside may be beneficial.[7]

7. Gender-affirming perioperative care to effectively mitigate further gender dysphoria and harmful healthcare discrimination experiences. The foundation of gender-affirming care includes self-identification, gender-identity fields, review of past medical and surgical history, perioperative testing and planning, and psychosocial issues.[5,7,8]

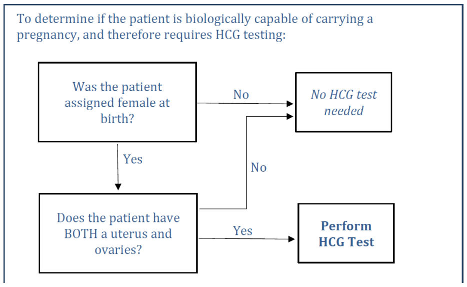

Figure S1: Gender-Affirming Chest Reconstruction ERAS pathway.

Intraoperative[14–22]

1. Induction
   - Standard American Society of Anesthesiology (ASA) monitors, electrocardiogram (ECG) leads placed posteriorly.
   - Consider SedLine or Bispectral Index (BIS) to guide depth of anesthetic and avoid intraoperative hypotension.
   - Anesthetic induction with Propofol. Consider Remifentanil bolus (1-2 mcg/kg) titrated to effect.
   - General Anesthesia with endotracheal tube or laryngeal mask airway (LMA). Consider using subglottic airway device with decompression ability.
   - Muscle relaxant not required by surgeon.
   - Orogastric tube (OGT) placement to decompress gastric contents.
   - Antimicrobial prophylaxis: betadine skin prep, antibiotics per clinical guidelines (typically single dose of cefazolin or appropriate antibiotic coverage before incision)
   - Prevent intraoperative hypothermia. Maintain core body temperature over 36 degrees Celsius.[16] Consider using lower body warming blanket.

2. Positioning
   - Supine. Arms out, shoulders even, pressure points padded, wrapped with kerlix
   - Consider inflatable air mattress (HoverMatt) for Body mass index (BMI) >35
   - Patient will be placed in seated position briefly during procedure after skin closure to determine nipple placement.
   - Sequential compression boots for deep vein thrombosis (DVT) prophylaxis

3. Maintenance
   - Aim for Total intravenous anesthesia (TIVA).[16,23] Start at >125 mcg/kg/min and titrate to maintain hemodynamic stability and adequate BIS (40s) or SedLine Patient State Index (PSI) 25-50.
   - TIVA: Propofol infusion +/-Remifentanil infusion: 0.1-0.2 mcg/kg/minute titrated.
   - Propofol infusion +/- inhalation agent. Consider adding Sevoflurane or Desflurane if increasing BIS/SedLine PSI.

4. Bleeding/Hematoma Prophylaxis
   - Intravenous Tranexamic acid (TXA) 30 mg/kg bolus (max 2000mg) over 20 minutes followed by infusion 10mg/kg/hour.[24,25]

5. Multimodal Pain Regimen
    - Intravenous Acetaminophen
    - Hydromorphone loaded prior to incision and titrated prior to emergence.
    - Adjuncts – Consider administering one of the following:
        - Diazepam 2.5mg
        - Dexmedetomidine 0.25-0.5 mcg/kg at the beginning of procedure. Can repeat as needed at the end of procedure. For same day discharge patients, please consider that dexmedetomidine may delay discharge.
        - Avoid Ketorolac per surgeon's request.
    - Local Anesthetics:
        - Surgeon administers Lidocaine and Epinephrine before surgical incision
        - Surgeon may infiltrate pectoralis and serratus fascia with Bupivacaine and Epinephrine before closure
        - Surgeon injects plain Bupivacaine through drains before extubation

6. Postoperative nausea vomiting (PONV) Prophylaxis
    - Dexamethasone
    - Ondansetron
    - Consider scopolamine if history of PONV or motion sickness.
    - Consider Haldol as rescue medication if no contraindications (cardiac meds, prolonged QTc interval).

7. Fluid Therapy
    - Balanced crystalloid administration.
    - Over resuscitation or under resuscitation should be avoided.
        - Typically, 1-2L lactated ringers for case
    - No Foley Catheter
        - Consider straight catheter if greater than 2L of fluid given or prominent bladder at the end of procedure.

8. Emergence
    - Consider sugammadex for muscle relaxant reversal.
    - To decrease the risk of hematoma formation, minimal coughing is ideal. Consider deep extubation when appropriate.

Disposition

1. Post anesthesia care unit (PACU) then home (same day surgery)
    - Majority of patients will be discharged same day.

2. Extended Stay (EXTD)
    - Patients with BMI>35
    - Other medical comorbidities

3. Counsel transmasculine patients about contraception if planning to administer sugammadex.

OTHER SURGICAL CONSIDERATIONS	
Goal for blood pressure at or above baseline after mastectomy	• Allows surgery team to assess hemostasis before closing and avoid risk of hematoma formation<br>• Temporarily elevate blood pressure to preoperative baseline with titrated doses of phenylephrine or ephedrine<br>• Communicate with surgeon for timing
Position Changes During Surgery	• Patients occasionally placed in sitting position after skin closure of mastectomy to determine nipple location<br>• Remifentanil bolus (0.5-1mcg/kg) and/or Propofol bolus prior to sitting to prevent coughing[26,26]
Dressings and Drains	• Jackson Pratt (JP) Drain x 2:<br>Plain Bupivacaine injected by surgeon into drain and then clamped. Once in PACU, drain unclamped by surgery team.<br>• Xeroform Bolster<br>• Purpose of bolster is to reduce the sheer forces between the underlying tissue and the graft to allow proper adherence of nipple graft and establish blood supply<br>• Bolster is sutured in place to reduce risk of nipple graft shifting in any way that inhibits healing<br>• Compression foam and vest applied prior to transport to PACU

Regional Anesthesia	• Not standard of care at institution<br>• For chronic pain patients, please consult with Regional Anesthesia and Acute Pain teams
**POSTOPERATIVE CARE**	
Complications	• Complications include hematoma which can necessitate emergency re-operation for wound exploration and hematoma evacuation<br>• These patients may have full stomachs and require RSI for intubation<br>• Pain management<br>• PONV management
Discharge Requirements	• Patients discharged home on Postoperative Day (POD) 0 unless otherwise clinically indicated (elevated BMI, comorbidities, long distance to travel)<br>• Postop physical exam in PACU<br>• No evidence of hematoma and pain is well controlled<br>• Patient is provided with number for surgery resident on call for all after-hours questions<br>• Also given number for anesthesia to contact with concerns
**POSTOPERATIVE FOLLOW-UP CARE**	
1 Week Post-op Visit	• Vest/compression foam/nipple areolar complex (NAC)bolster removal and assessment of skin graft<br>• Drain removal as well if drain output is <20 cc for 2 consecutive days<br>• Review daily dressing care<br>• Assessment of pain management<br>• Education on activity restrictions
1 Month Post-op Visit	• Begin scar management with daily scar massage<br>• Usually discontinue compressions vest at this time

	• Counsel patients to continue to refrain from heavy lifting or strenuous physical activity for additional two weeks<br>• They may gradually return to working out and overhead reaching at 6 week postop mark
3-4 Month Post-op Visit	• Brief visit to check in regarding scar management/chest contour
1 Year	• Annual photos

REFERENCES

1. Oles N, Ganor O, Aquino NJ, Boskey ER. Surgical Affirmation for Gender-Diverse Youth. *Journal of Pediatric Surgical Nursing*. 2021;10(1):32-39. doi:10.1097/JPS.0000000000000279

2. Roque RA. Transgender pediatric surgical patients—Important perioperative considerations. Thomas M, ed. *Pediatr Anaesth*. 2020;30(5):520-528. doi:10.1111/pan.13845

3. Kim E, Mukerji S, Debryn D, Price R, Streed C, Nozari A. Oxygen desaturation in a transgender man: initial concerns and recommendations regarding the practice of chest binding: a case report. *J Med Case Reports*. 2022;16(1):333. doi:10.1186/s13256-022-03527-z

4. Discepolo K, Aquino N. Considerations for Transgender Patients Requiring Dental Rehabilitation. *J Dent Child (Chic)*. 2022;89(1):46-51.

5. Tollinche LE, Van Rooyen C, Afonso A, Fischer GW, Yeoh CB. Considerations for Transgender Patients Perioperatively. *Anesthesiology Clinics*. 2020;38(2):311-326. doi:10.1016/j.anclin.2020.01.009

6. Tollinche LE, Walters CB, Radix A, et al. The Perioperative Care of the Transgender Patient: *Anesthesia & Analgesia*. 2018;127(2):359-366. doi:10.1213/ANE.0000000000003371

7. Aquino NJ, Ganor O, Chrisos HA, Oles N, Boskey ER. Perioperative Issues With Gender-Diverse Youth. *Journal of Pediatric Surgical Nursing*. 2021;10(1):23-31. doi:10.1097/JPS.0000000000000282

8. Reece-Nguyen TL, Tollinche L, Van Rooyen C, Roque RA. Current challenges faced by transgender and gender-diverse patients and providers in anesthesiology. *International Anesthesiology Clinics*. 2023;61(1):26-33. doi:10.1097/AIA.0000000000000384

9. Shah S, Khanna P, Bhatt R, Goyal P, Garg R, Chawla R. Perioperative anaesthetic concerns in transgender patients: Indian perspective. *Indian J Anaesth*. 2019;63(2):84. doi:10.4103/ija.IJA_640_18

10. Boskey ER, Johnson JA, Harrison C, et al. Ethical Issues Considered When Establishing a Pediatrics Gender Surgery Center. *Pediatrics*. 2019;143(6):e20183053. doi:10.1542/peds.2018-3053

11. Coleman E, Bockting W, Botzer M, et al. Standards of Care for the Health of Transsexual, Transgender, and Gender-Nonconforming People, Version 7. *International Journal of Transgenderism*. 2012;13(4):165-232. doi:10.1080/15532739.2011.700873

12. Aquino NJ, Boskey ER, Staffa SJ, et al. A Single Center Case Series of Gender-Affirming Surgeries and the Evolution of a Specialty Anesthesia Team. *JCM*. 2022;11(7):1943. doi:10.3390/jcm11071943

13. Coleman E, Radix AE, Bouman WP, et al. Standards of Care for the Health of Transgender and Gender Diverse People, Version 8. *International Journal of Transgender Health*. 2022;23(sup1):S1-S259. doi:10.1080/26895269.2022.2100644

14. Astanehe A, Temple-Oberle C, Nielsen M, et al. An Enhanced Recovery after Surgery Pathway for Microvascular Breast Reconstruction Is Safe and Effective: *Plastic and Reconstructive Surgery - Global Open*. 2018;6(1):e1634. doi:10.1097/GOX.0000000000001634

15. Chiu C, Aleshi P, Esserman LJ, et al. Improved analgesia and reduced post-operative nausea and vomiting after implementation of an enhanced recovery after surgery (ERAS) pathway for total mastectomy. *BMC Anesthesiol*. 2018;18(1):41. doi:10.1186/s12871-018-0505-9

16. Temple-Oberle C, Shea-Budgell MA, Tan M, et al. Consensus Review of Optimal Perioperative Care in Breast Reconstruction: Enhanced Recovery after Surgery (ERAS) Society Recommendations. *Plastic and Reconstructive Surgery*. 2017;139(5):1056e-1071e. doi:10.1097/PRS.0000000000003242

17. Offodile AC, Gu C, Boukovalas S, et al. Enhanced recovery after surgery (ERAS) pathways in breast reconstruction: systematic review and meta-analysis of the literature. *Breast Cancer Res Treat*. 2019;173(1):65-77. doi:10.1007/s10549-018-4991-8

18. Tan YZ, Lu X, Luo J, et al. Enhanced Recovery After Surgery for Breast Reconstruction: Pooled Meta-Analysis of 10 Observational Studies Involving 1,838 Patients. *Front Oncol*. 2019;9:675. doi:10.3389/fonc.2019.00675

19. Voigt M, Fröhlich CW, Waschke KF, Lenz C, Göbel U, Kerger H. Prophylaxis of postoperative nausea and vomiting in elective breast surgery. *Journal of Clinical Anesthesia*. 2011;23(6):461-468. doi:10.1016/j.jclinane.2011.01.005

20. Kennedy GT, Hill CM, Huang Y, et al. Enhanced recovery after surgery (ERAS) protocol reduces perioperative narcotic requirement and length of stay in patients undergoing mastectomy with implant-based reconstruction. *The American Journal of Surgery*. 2020;220(1):147-152. doi:10.1016/j.amjsurg.2019.10.007

21. Sindali K, Harries V, Borges A, et al. Improved patient outcomes using the enhanced recovery pathway in breast microsurgical reconstruction: a UK experience. *JPRAS Open*. 2019;19:24-34. doi:10.1016/j.jpra.2018.10.002

22. Cuccolo NG, Kang CO, Boskey ER, et al. Masculinizing Chest Reconstruction in Transgender and Nonbinary Individuals: An Analysis of Epidemiology, Surgical Technique, and Postoperative Outcomes. *Aesth Plast Surg*. 2019;43(6):1575-1585. doi:10.1007/s00266-019-01479-2

23. Persing S, Manahan M, Rosson G. Enhanced Recovery After Surgery Pathways in Breast Reconstruction. *Clinics in Plastic Surgery*. 2020;47(2):221-243. doi:10.1016/j.cps.2019.12.002

24. Goobie SM, Staffa SJ, Meara JG, et al. High-dose versus low-dose tranexamic acid for paediatric craniosynostosis surgery: a double-blind randomised controlled non-inferiority trial. *British Journal of Anaesthesia*. 2020;125(3):336-345. doi:10.1016/j.bja.2020.05.054

25. Goobie SM, Faraoni D. Tranexamic acid and perioperative bleeding in children: what do we still need to know? *Current Opinion in Anaesthesiology*. 2019;32(3):343-352. doi:10.1097/ACO.0000000000000728

26. Son HW, Lee JM, Park SH, Lee YJ, Oh JM, Hwang SK. Fentanyl versus Remifentanil for Cough Suppression and Recovery after Video-Assisted Thoracic Surgery. *J Chest Surg*. 2021;54(3):200-205. doi:10.5090/jcs.20.136

Document Attributes

**Title**	Enhanced Recovery after Surgery for Gender Affirming Chest Reconstruction Surgery		
**Author**	Whitney Roberts, CRNA; Kristin Gemmill, CRNA; Alyson Crest, CRNA; Bistra Vlassakova, MD Nelson Aquino, DNP,CRNA	**Date of Origin**	February 2021
**Reviewed/ Revised by**	Sabeena Chacko,MD/ Nelson Aquino, DNP,CRNA	**Dates Reviewed/Revised**	March 2021 March 2023
**Copyright**	©Boston Children's Hospital, 2021	**Last Modified**	March 2023
**Approved**	Sabeena Chacko, MD		
